# Peripheral Fragile X messenger ribonucleoprotein is required for the timely closure of a critical period for neuronal susceptibility in the ventral cochlear nucleus

**DOI:** 10.3389/fncel.2023.1186630

**Published:** 2023-05-25

**Authors:** Xiaoyan Yu, Yuan Wang

**Affiliations:** Program in Neuroscience, Department of Biomedical Sciences, Florida State University College of Medicine, Tallahassee, FL, United States

**Keywords:** Fragile X syndrome, critical period, sensory organ, afferent influence, hearing onset, brain development

## Abstract

Alterations in neuronal plasticity and critical periods are common across neurodevelopmental diseases, including Fragile X syndrome (FXS), the leading single-gene cause of autism. Characterized with sensory dysfunction, FXS is the result of gene silencing of *Fragile X messenger ribonucleoprotein 1 (FMR1)* and loss of its product, Fragile X messenger ribonucleoprotein (FMRP). The mechanisms underlying altered critical period and sensory dysfunction in FXS are obscure. Here, we performed genetic and surgical deprivation of peripheral auditory inputs in wildtype and *Fmr1* knockout (KO) mice across ages and investigated the effects of global FMRP loss on deafferentation-induced neuronal changes in the ventral cochlear nucleus (VCN) and auditory brainstem responses. The degree of neuronal cell loss during the critical period was unchanged in *Fmr1* KO mice. However, the closure of the critical period was delayed. Importantly, this delay was temporally coincidental with reduced hearing sensitivity, implying an association with sensory inputs. Functional analyses further identified early-onset and long-lasting alterations in signal transmission from the spiral ganglion to the VCN, suggesting a peripheral site of FMRP action. Finally, we generated conditional *Fmr1* KO (cKO) mice with selective deletion of FMRP in spiral ganglion but not VCN neurons. cKO mice recapitulated the delay in the VCN critical period closure in *Fmr1* KO mice, confirming an involvement of cochlear FMRP in shaping the temporal features of neuronal critical periods in the brain. Together, these results identify a novel peripheral mechanism of neurodevelopmental pathogenesis.

## Introduction

A fundamental feature of brain development is the presence of sensitive periods during which certain neuronal properties are particularly susceptible to changes in afferent inputs or sensory experience (Hensch, 2004; Knudsen, 2004; Buran et al., 2014; Anbuhl et al., 2022). A sensitive period with distinct temporal signatures, i.e., rapid onset and/or closing, is often referred as a critical period (Hensch, 2005). Proper closure of critical periods is considered neuroprotective for brain development (Takesian and Hensch, 2013). Alterations in critical periods and experience-dependent brain plasticity are commonly found in neurodevelopmental disorders such as autism and schizophrenia (LeBlanc and Fagiolini, 2011; Takesian and Hensch, 2013; Do et al., 2015). However, the pathogeny and progression of critical period-related properties under disease conditions are poorly understood.

We investigated these topics by investigating Fragile X syndrome (FXS), a leading monogenetic cause of intellectual disability and autism (Santoro et al., 2012; Hagerman et al., 2017). FXS is caused by transcriptional silencing of the *FMR1* gene and the resultant loss of its product, Fragile X messenger ribonucleoprotein (FMRP). Individuals with FXS display prominent sensory dysfunction including acoustic hyperactivity and compromised hearing in noise (Rotschafer et al., 2015; McCullagh et al., 2020). In developing sensory systems, deprivation or extended exposure to certain sensory stimuli results in sensory map reorganization. Studies using *Fmr1* knockout (KO) mice report that this sensory remapping is diminished in the visual and auditory cortex but remains unchanged in the somatosensory cortex (Dolen et al., 2007; Harlow et al., 2010; Kim et al., 2013; Zhong et al., 2018). FMRP loss-induced alterations in experience-dependent plasticity of ion channels, synaptic transmission, dendritic maturation, and oscillation, have also been reported across brain regions (Strumbos et al., 2010; Sidorov et al., 2013; Doll and Broadie, 2015, 2016; Doll et al., 2017; Kissinger et al., 2020; Golovin et al., 2021). Intriguingly, one study revealed a shift in the time window of the critical period for long-term synaptic potentiation instead of diminishing the plasticity entirely (Harlow et al., 2010). Thus, the underlying mechanism of FMRP-regulated neuronal critical period is circuit- and cell type-specific.

**FIGURE 1:**
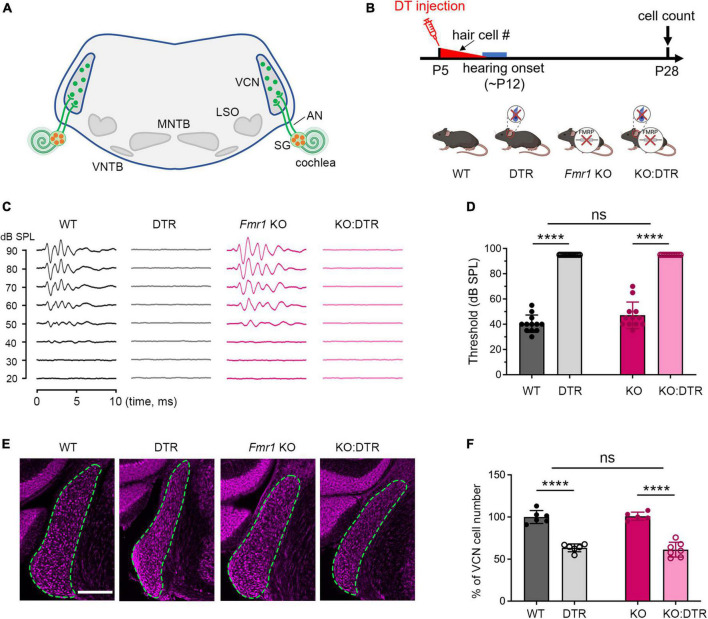
Hair cell deletion-induced afferent deprivation before hearing onset resulted in significant neuronal loss in the VCN in both WT and *Fmr1* KO mice. **(A)** Schematic diagram of the projection from the SG in the cochlea to the VCN in the brainstem. **(B)** Experimental timeline and four experimental groups (WT, DTR, *Fmr1* KO, and KO:DTR). DT or saline (as control) was administered at P5, leading to complete hair cell loss before hearing onset. Tissue collection and data analysis were conducted at P28. **(C)** Examples of ABR patterns in each experimental group. The injection was performed at P5 or P14 and ABRs were recorded at P28-35 in response to click stimuli. **(D)** ABR thresholds in response to click stimulus in WT (*n* = 12), DTR (*n* = 14), *Fmr1* KO (*n* = 12), and KO:DTR (*n* = 13) groups at P28 following DT/saline injection at P5. DTR and KO:DTR groups failed to produce detectable ABRs to click stimulus at 90 dB. **(E)** NeuroTrace staining in the VCN (dashed circles) of each experimental group. **(F)** Quantitative analysis of the neuron number in the VCN in the four experimental groups at P28. Hair cell deletion led to significant neuronal loss in both DTR (*n* = 6, *p* < 0.0001) and KO:DTR (*n* = 7, *p* < 0.0001) groups as compared to WT (*n* = 6) and *Fmr1* KO (*n* = 5) control groups, respectively (two-way ANOVA followed by Tukey’s *post hoc* tests). There was no significant interaction between *Fmr1* genotype and afferent condition (*p* = 0.588). Scale bar = 250 μm in panel **(E)**. AN, auditory nerve; LSO, lateral superior olive; MNTB, medial nucleus of the trapezoid body; SG, spiral ganglion; VCN, ventral cochlear nucleus; VNTB, ventral nucleus of the trapezoid body; #, number; ****p < 0.0001; ns, not significant.

In this study, we aimed to identify the specific aspects of critical periods that are affected in the auditory system of *Fmr1* KO mice. Proper development of the central auditory system depends on afferent inputs (both spontaneous and sensory-driven activities) from cochlear hair cells via the spiral ganglion (SG, Figure 1A) (Friauf and Lohmann, 1999; Rubel and Fritzsch, 2002; Kandler et al., 2009; Takesian et al., 2009; Bailey and Green, 2014). One well-characterized critical period is neuronal loss in the ventral cochlear nucleus (VCN) upon deprivation of afferent inputs from the SG. In mice, substantial neuronal death occurs when afferent deprivation is induced at or before postnatal day (P) 11 but not at or after P14 (Mostafapour et al., 2000; Tong et al., 2015), providing a sensitive readout for studying critical period dynamics. Using both genetic and surgical approaches to block afferent inputs from the cochlea to the brain, we found that *Fmr1* KO mice exhibited a delay in the closure of the critical period for deafferentation-induced neuronal loss in the VCN, without affecting the degree of neuronal death within the critical period.

We next examined how *Fmr1* KO affects the onset of auditory experience and identify its relationship to the critical period closure in the VCN. Hearing onset, is considered a key milestone for a wide range of developmental events in the auditory system (Kraus and Aulbach-Kraus, 1981; Magnusson et al., 2005; Sonntag et al., 2011; Kim and Rutherford, 2016), including the critical period closure in the VCN. Remarkably, we observed reduced hearing sensitivity during the same period of the delay in the critical period closure and long-lasting alterations in the SG to VCN signal transmission in *Fmr1* KO mice. Finally, to identify the potential sites of FMRP action in shaping the critical period, we developed a conditional *Fmr1* KO mouse line with selective FMRP knockout in the SG but not in the VCN. We identified a contribution of FMRP loss from the SG to the delayed critical period closure for VCN neuronal susceptibility.

## Materials and methods

### Animals

All animal procedures were approved by the Florida State University Institutional Animal Care and Use Committee and performed in accordance with the National Institutes of Health Guide for the Care and Use of Laboratory Animals. For all experiments, the day of birth was defined as postnatal day 0 (P0). Oligos used for PCR-based genotyping of mouse lines are listed in Table 1.

**TABLE 1:** DNA oligos used in genotyping.

Gene	Primers	Sequences (from 5’′ to 3′)	Diagnostic size
DTR	*Pou4f3* forward	CAC TTG GAG CGC GGA GAG CTA G	WT: none DTR: ∼150 bp
	*Pou4f3* reverse	CCG ACG GCA GCA GCT TCA TGG TC	
*Fmr1* loxp	*Fmr1* 2f	GTT GAG CGG CCG AGT TTG TCA G	WT: 120 bp Male cKO: 220 bp Female heterozygote: 120 bp and 220 bp Female cKO homozygote: 220 bp
	*Fmr1* 3r	CCC ACT GGG AGA GGA TTA TTT GGG	
CR-iCre	19820	AGG TCT GGG AAG GAG TGT CA	Mutant: 175 bp Heterozygote: 175 bp and 125 bp WT: 125 bp
	19821	CCA CTA GAT CGA ATT CCG AAG	
	9485	ACC TGG AGA TTG TGC TCT GC	

#### *Pou4f3^DTR/+^* mice

Mouse breeders for wildtype (WT; C57BL/6J), *Fmr1* KO (B6.129P2-*Fmr*1^*TM*1*Cgr*^/J), and *Pou4f3^DTR/+^* (B6.Cg-*Pou4f3^*TM*1^.^1(HBEGF)Jsto^*/RubelJ) were purchased from the Jackson Laboratory (Bar Harbor, MA, USA) and bred at Florida State University. The *Pou4f3^DTR/+^* mouse is a transgenic model in which the human diphtheria toxin (DT) receptor (hDTR) is selectively expressed in hair cells driven by the promoter of *Pou4f3*, which encodes a hair cell specific transcription factor (Golub et al., 2012; Mahrt et al., 2013; Kaur et al., 2015; Tong et al., 2015). A systemic injection of DT leads to specific deletion of hair cells without damaging other structures in the cochlea. Heterozygote male *Pou4f3^DTR/+^* mice were crossed with either WT or *Fmr1* KO female mice. The resultant *Fmr1* KO:*Pou4f3^DTR/+^* male pups from *Fmr1* KO dams were then crossed with *Fmr1* KO females. This breeding paradigm produced four offspring genotypes: *Pou4f3^DTR/+^* (DTR), *Pou4f3*^+/+^ (WT), *Fmr1* KO:*Pou4f3^DTR/+^* (KO:DTR), and *Fmr1* KO:*Pou4f3*^+/+^ (*Fmr1* KO) (Figure 1B). Mice of either sex from these offspring were used in this study.

#### *Fmr1* conditional knockout (cKO) mice

*Fmr1^loxp^* mice on the C57BL/6J background were obtained from Dr. David Nelson at Baylor College of Medicine (Mientjes et al., 2006). CR-iCre (B6(Cg)-Calb2^*TM*1(cre)*Zjh*^/J) mice were purchased from the Jackson Laboratory. This mouse line has cre recombinase expression to calretinin (CR)-expressing neurons, driven by the endogenous *Calb2* promoter/enhancer elements (Taniguchi et al., 2011). Homozygote female *Fmr1^loxp/loxp^* mice were crossed with heterozygote male CR-iCre mice. Their male offspring were either *Fmr1^loxp/y^*:CR-iCre (cKO) or *Fmr1^loxp/y^* without CR-iCre (control); both were used in this study. The female offspring were heterozygous for *Fmr1* KO and not used in this study.

### Diphtheria toxin (DT) administration

Diphtheria toxin (DT) powder was purchased from List Biological Laboratories (Campbell, CA, USA) and dissolved in 0.9% sterile saline at 2 mg/ml of stock concentration and stored at −20°C. Stock DT solution was further diluted into 1 μg/ml of working concentration before use. Neonatal mice received a single dose of either DT injection at the dosage of 5 ng/g body weight or 0.9% saline injection at P5 or P14 (Tong et al., 2015). The injection was made in the thigh muscle of the right hind leg. Following either saline or DT injection, mice were allowed to survive to the age of P27–35 before tissue collection.

### Auditory brainstem responses: procedure and analyses

Auditory brainstem responses (ABRs) were recorded to (1) confirm the effect of DT administration before tissue collection and (2) determine alterations in hearing sensitivity of *Fmr1* KO and cKO mice across ages. Animals were anesthetized with an intraperitoneal injection of 105 mg/kg ketamine and 9 mg/kg xylazine and a supplemental dose of 20 mg/kg ketamine if needed. Mice were then placed on a heating pad to maintain their body temperature during ABR recordings. Recording was conducted in a sound-attenuating chamber using an ABR acquisition system (Tucker Davis Technologies; Alachua, FL, USA). Subdermal needle electrodes (Rochester Electro-Medical; Lutz, FL, USA) were used for recording, with the positive electrode positioned at the vertex of the skull, the reference electrode below the pinna of the right ear, and the ground electrode in the right thigh. A multi-field speaker (MF1; Tucker Davis Technologies) was placed 10 cm from the right ear for open field recording. Click (0.1 ms in duration) and tone bursts of 4, 8, 16, 24, and 32 kHz (5 ms in duration) were presented at a rate of 21/s.

Calibration was performed using an Etymotic (Fort Worth, TX, USA) low noise probe and microphone (ER10B +) and 1/4” free field measure calibration mic kit (PCB-378C01; PCB Piezotronics; Depew, NY, USA). Each type of stimuli (click and each frequency of tone) was presented at levels from 90 dB sound pressure level (SPL) to 20 dB SPL in 10 dB decrements first and then repeated in 5 dB decrements at levels near the threshold. Biological responses were pre-amplified (100×; Mdeusa4Z Pre-amp/Digitizer and Headstage; Tucker Davis Technologies), bandpass filtered (300–3,000 Hz), digitized (RZ6-A-P1 bioacoustic system; Tucker Davis Technologies), and averaged from a total of 512 trials over a 20 ms window using the BioSigRZ software (Tucker Davis Technologies).

For examining the efficiency of DT injection, animals with DT or saline injection at P5 or P14 were recorded at P27–35. Click stimulus was presented to confirm DT-induced hearing loss. For examining hearing sensitivity in *Fmr1* KO and cKO mice, animals were recorded at P14, P19, and P60. Both click stimulus and tone bursts were used to identify changes in ABR threshold and peak latency. ABR threshold was defined as the lowest SPL that evokes a detectable and repeatable response (Song et al., 2006). If no ABR wave was detected at 90 dB for a specific stimulus, a nominal threshold of 95 dB was assigned. Peak latencies were measured for the first three peaks at P14 and the first four peaks at P19 and P60 in response to click stimulus and tone bursts of 8, 16, and 32 kHz at 90 dB SPL.

### Cochlea ablation

Unilateral cochlea ablation (left cochlea) was performed in WT and *Fmr1* KO mice at P14 and P19 as well as in *Fmr1* cKO mice and their littermate controls at P14. Animals were anesthetized either with an intraperitoneal injection of 70–105 mg/kg ketamine and 6–9 mg/kg xylazine, or via isoflurane inhalation. Cochlea ablation was performed as described previously (Mostafapour et al., 2000). Briefly, the tympanic membrane was penetrated to access the middle ear and the basal turn of the cochlea was exposed after the ossicles were removed. The bony cochlear structure was penetrated using a 25-gauge needle and cochlear content was aspirated using a fine glass pipette. The modiolus was destroyed with a pair of fine tweezers. The skin incision was closed with tissue glue. Analgesia was given with subcutaneous injections of buprenorphine at 0.05 mg/kg of body weight. Animals were allowed to survive for 14–18 days following the surgery. A complete cochlea ablation was confirmed visually by substantial damage to the spiral structure of the cochlea under an operating microscope after tissue collection.

### Brain tissue processing and immunocytochemistry

Animals were deeply anesthetized with an intraperitoneal injection of ketamine and xylazine and transcardially perfused with 0.9% saline followed by 4% paraformaldehyde (PFA) in 0.1 M phosphate buffer (PB), pH 7.4. Brains were removed from the skull, post-fixed in 4% PFA overnight at 4°C, and transferred to 30% sucrose in PB for cryoprotection. Brains were sectioned coronally at 40 μm using a freezing sliding microtome and collected in 0.01 M phosphate-buffered saline (PBS), pH 7.4. One in every four sections were incubated with primary antibody solutions diluted in PBS with 0.3% Triton X-100 and 5% normal goat serum overnight at room temperature. After washing in PBS, sections were incubated with Alexa Fluor secondary antibodies in PBS at 1:500 for 4 hours at room temperature. Nuclear and cell body counterstains were performed together with the incubation of secondary antibodies using DAPI (1:1,000) and NeuroTrace 640/660 (1:1,000; Thermo Fisher Scientific; RRID:AB_2572212). For cell counting and cell size measurement, sections were incubated with DAPI and NeuroTrace 640/660 without antibody immunostaining. After washing, sections were mounted on gelatin-coated slides and coverslipped with Fluoromount-G mounting medium (Southern Biotech, Birmingham, AL, USA).

Primary antibodies used include: rabbit anti-FMRP (1:100; Cell Signaling Technology; Danvers, MA; RRID:AB_10950502), chicken anti-CR (1:1,000; EnCor Biotechnology; Gainesville, FL; RRID:AB_2572241), and mouse anti-TuJ1 (1:500; R&D Systems; Minneapolis, MN; RRID:AB_357520). Secondary antibodies were purchased from Thermo Fisher Scientific (Waltham, MA), including goat anti-rabbit 488 (RRID:AB_143165), goat anti-chicken 568 (AB_2534098), and goat anti-mouse 660 (AB_2535723).

### Cochlea tissue processing and immunocytochemistry

To confirm the efficiency and specificity of FMRP deletion in cKO mice, cochlea tissues were collected from cKO and their littermate control mice at P14 and P28. Following transcardial perfusion, temporal bones were isolated, round and oval windows were opened, and a small hole was made in the apex of the cochlea to facilitate fixer solution infiltration. Cochleae were then post-fixed in 4% PFA overnight at 4°C, washed in PBS, and decalcified in 10% ethylenediaminetetraacetic acid for 2 days at 4°C. Cochleae were then embedded following a previously described protocol (Coleman et al., 2009). Briefly, decalcified cochleae were transferred to 10% sucrose for 1 h at room temperature, and then to 15% sucrose overnight at 4°C, and finally to 1:1 solution of 15% sucrose and optimal cutting temperature (OCT) compound (Fisher Healthcare) overnight at 4°C. Tissues were then embedded in OCT compound for cryosectioning. Cochleae were sectioned along the modiolus direction at 20 μm thickness and mounted on gelatin-coated slides. For immunostaining, sections were washed in PBS for 10 minutes at 37°C to remove OCT and incubated with primary antibody solutions diluted in PBS with 0.3% Triton X-100 and 5% normal goat serum overnight at 4°C. After washing in PBS, sections were incubated with Alexa Fluor secondary antibodies in PBS at 1:500 for 4 h at room temperature. Sections were washed in PBS and coverslipped with Fluoromount-G mounting medium.

### Cell counting and size measurement in the VCN

One-in-four series of sections that contain the VCN including the posterior VCN and anterior VCN (AVCN) were used for cell counting using the “cell counter” plugin in the ImageJ software (National Institutes of Health). Images were captured with a 60× oil objective attached to an Olympus FV-1200 confocal microscope. Cells with an identifiable cytoplasmic and nuclear boundary and a visible nucleolus were counted based on NeuroTrace staining. Cells with the cross-sectional cell body size <50 μm^2^ were excluded to minimize glial cell counts. The total neuron number of the VCN was calculated as the sum of neuron counts from all VCN-containing sections and normalized to the mean total neuron number of the control counterparts (either the control animals or the intact side of the same animals) within the same experiment. The VCN neuron number is presented as a percentage with controls as 100% on average in the Results and figures.

Neuronal cell size was measured from one AVCN-containing section selected from each side of each animal. The selection of section followed the criteria as described in Tierney et al. (1997), based on the location about 25–40% along the rostral-to-caudal axis of the VCN. Within each section, neurons within the AVCN and with a clear cytoplasmic boundary, a well-defined nucleus and visible nucleolus were selected, and their cross-sectional somatic areas were measured in ImageJ. Cells with the cross-sectional cell body size <50 μm^2^ were excluded to minimize glial cells. At least 50 neurons were measured from each animal.

### Quantitative analysis of FMRP and CR immunostaining in *Fmr1* cKO mice

The efficiency and specificity of FMRP deletion were examined in the spiral ganglion and VCN of cKO mice and their littermate controls at P14 and 28. Cochlea and brain sections were tri-labeled with FMRP, CR, and NeuroTrace (or Tuj1 on some cochlea sections). Images were captured with 20× air or 60× oil objectives attached to the FV-1200 confocal microscope. Quantification was conducted with 60× images without further image processing. For each animal, 4–6 cochlea sections containing SG neurons (SGNs) and one brainstem section containing a major body of the AVCN were chosen for quantification. Within each cell group, the total neuron number was counted based on NeuroTrace or TuJ1 staining. Then, the numbers of neurons that were CR-immunoreactive (CR+), FMRP-immunoreactive (FMRP+), or both were counted separately. Finally, the percentages of CR+ and FMRP+ neurons among all neurons in the cell group were calculated, as well as the percentage of FMRP+ neurons among CR+ neurons.

### Experimental design and statistical analyses

In all experiments, at least three animals per genotype and age were used for each analysis. Quantitative measures were performed by two individual investigators with blind data collection. One individual animal was used as one individual data point. Statistical analyses were conducted using GraphPad Prism software (GraphPad, San Diego, CA, USA). Two-tailed, unpaired Student’s *t*-test was used to compare between *Fmr1* genotypes. Parametric and non-parametric tests were used for data that pass and fail Shapiro–Wilk normality test, respectively. Two-way ANOVA or repeated measures two-way ANOVA followed by Tukey’s *post-hoc* multiple comparisons were used to determine the effects of *Fmr1* genotype and afferent condition on neuron number and neuronal size, as well as the effect of *Fmr1* genotype on ABR properties (threshold and latency) across stimuli. Because several data sets for ANOVA analyses fail the normality test, we additionally performed non-parametric *t*-tests between age-matched control and *Fmr1* KO groups and found that the two tests revealed the same conclusions (see Tables 3–6). Thus, ANOVA results were presented in the figures and Results for consistency. Sample sizes are given in figure legends or tables. Significance was determined by *p* < 0.05. Data are given as mean ± standard deviation (SD) for consistency as most of the data sets fit a normal distribution. For illustration, image brightness, contrast, and gamma were adjusted using Adobe Photoshop (Adobe System, Mountain View, CA, USA). Schematics were created with BioRender.com.

## Results

### *Fmr1* KO did not affect the degree of afferent deprivation-induced neuronal loss within a critical period

Afferent deprivation at early postnatal ages leads to neuronal cell death in mice (Mostafapour et al., 2000; Tong et al., 2015). To investigate how FMRP absence impacts this influence, DTR mice were used to induce afferent deprivation. In this mouse line, human DT receptor is selectively expressed in hair cells (driven by *Pou4f3* promoters) so that a systemic injection of DT leads to specific deletion of hair cells without damaging other structures in the cochlea (Golub et al., 2012; Mahrt et al., 2013; Kaur et al., 2015; Tong et al., 2015). DTR mice were crossed with WT or *Fmr1* KO mice to generate four offspring genotypes: DTR, WT, KO:DTR, and *Fmr1* KO. The timelines, animal groups and analyses of experiments were summarized in Table 2.

**TABLE 2:** Timeline, genotype, and analysis for experiments.

**DT-induced afferent deprivation**	
• Genotypes • Age at DT or saline injection • Age at tissue collection • Analysis	DTR, *Fmr1* KO:DTR and their controls P5, P14 P28-P35 Cell count in the VCN; Cell size measurement in the AVCN; ABR test
**Unilateral cochlea ablation**	
• Genotypes • Age • Age at tissue collection • Analysis	(1) *Fmr1* KO and WT; (2) cKO and their control littermates P14 and P19 P28–P35 Cell count in the VCN
**ABR analysis**	
• Genotypes • Age • Analysis	(1) *Fmr1* KO and WT; (2) cKO and their control littermates P14, P19, and P60–63 ABR threshold; wave and interpeak latencies
**FMRP immunostaining and quantification**	
• Genotypes • Age • Regions	cKO and their control littermates P14-P28 Cochlea and brainstem

**TABLE 3:** Latencies of each wave and interpeak in response to click and tone stimuli in WT and *Fmr1* KO mice at P14.

Latency	Click			8 kHz			16 kHz			32 kHz		
	**WT** **(*n* = 16)**	***Fmr1* KO** **(*n* = 11)**	*p* *	**WT** **(*n* = 16)**	***Fmr1* KO** **(*n* = 11)**	***p***	**WT** **(*n* = 16)**	***Fmr1* KO** **(*n* = 11)**	***p***	**WT** **(*n* = 16)**	***Fmr1* KO** **(*n* = 7)**	***p***
Wave I	1.54 (0.09)	1.68 (0.15)	0.077	2.18 (0.12)	2.42 (0.25)	0.0005	1.73 (0.14)	1.95 (0.17)	0.0014	1.71 (0.17)	1.97 (0.19)	0.0017
Wave II	2.58 (0.17)	2.82 (0.16)	0.0478	3.20 (0.25)	3.59 (0.35)	0.0003	2.76 (0.25)	3.07 (0.23)	0.0051	2.75 (0.23)	3.00 (0.27)	0.1067
Wave III/IV	3.95 (0.27)	4.19 (0.28)	0.2525	4.42 (0.25)	5.02 (0.48)	<0.0001	4.08 (0.27)	4.42 (0.36)	0.0373	4.07 (0.31)	4.50 (0.51)	0.0196
Interpeak I-II	1.04 (0.11)	1.14 (0.11)	0.4381	1.02 (0.19)	1.17 (0.17)	0.0954	1.03 (0.16)	1.12 (0.13)	0.5544	1.05 (0.21)	1.03 (0.13)	0.9987 *0.7319*^#^
Interpeak II-III/IV	1.37 (0.14)	1.37 (0.15)	>0.9999	1.23 (0.17)	1.43 (0.24)	0.0394 *0.0137^#^*	1.32 (0.20)	1.35 (0.19)	0.9920	1.31 (0.22)	1.50 (0.29)	0.1462
Interpeak I-III/IV	2.42 (0.20)	2.51 (0.16)	0.7966	2.25 (0.17)	2.59 (0.29)	0.0012	2.35 (0.20)	2.47 (0.24)	0.6305	2.36 (0.28)	2.53 (0.36)	0.3854

*Adjusted *p* values of Tukey’s *post hoc* tests following two-way ANOVA.

Italic values with # are *p*-values of non-parametric t-tests between WT and *Fmr*1 KO mice.

Data are presented as mean (SD).

**TABLE 4:** Latencies of each wave and interpeak in response to click and tone stimuli in WT and *Fmr1* KO mice at P19.

Latency	Click			8 kHz			16 kHz			32 kHz		
	**WT** **(*n* = 10)**	***Fmr1* KO** **(*n* = 8)**	*p* *	**WT** **(*n* = 9)**	***Fmr1* KO** **(*n* = 8)**	***p***	**WT** **(*n* = 9)**	***Fmr1* KO (*n* = 8)**	***p***	**WT** **(*n* = 9)**	***Fmr1* KO** **(*n* = 8)**	***p***
Wave I	1.29 (0.06)	1.38 (0.05)	0.0146	1.73 (0.08)	1.81 (0.05)	0.0389	1.46 (0.07)	1.56 (0.06)	0.0048	1.35 (0.05)	1.47 (0.08)	0.0024 *0.0068^#^*
Wave II	2.39 (0.12)	2.47 (0.15)	0.7629 *0.2188^#^*	2.59 (0.20)	2.76 (0.15)	0.1045	2.28 (0.19)	2.46 (0.13)	0.0712	2.14 (0.11)	2.34 (0.17)	0.0440
Wave III	3.27 (0.21)	3.47 (0.12)	0.0693	3.69 (0.17)	3.89 (0.17)	0.0868	3.48 (0.12)	3.67 (0.17)	0.1013	3.21 (0.17)	3.50 (0.23)	0.0043
Wave IV	4.18 (0.35)	4.60 (0.26)	0.0151	4.37 (0.21)	4.80 (0.28)	0.0232 *0.0101^#^*	4.22 (0.21)	4.54 (0.20)	0.1846	4.10 (0.35)	4.43 (0.26)	0.1058
Interpeak I-II	1.10 (0.10)	1.09 (0.14)	0.9984	0.86 (0.15)	0.95 (0.14)	0.4358	0.82 (0.14)	0.90 (0.09)	0.5574 *0.0568^#^*	0.79 (0.08)	0.87 (0.11)	0.4693 *0.0740^#^*
Interpeak II-III	1.98 (0.15)	2.09 (0.11)	0.3659	1.10 (0.13)	1.13 (0.15)	0.9895	1.20 (0.22)	1.21 (0.13)	0.9997	1.07 (0.09)	1.17 (0.10)	0.5464
Interpeak III-IV	0.91 (0.16)	1.14 (0.18)	0.0522	0.70 (0.10)	0.91 (0.16)	0.0893	0.75 (0.14)	0.92 (0.18)	0.3092 *0.0821^#^*	0.89 (0.31)	0.92 (0.17)	0.9919
Interpeak I-IV	2.89 (0.29)	3.22 (0.25)	0.0441	2.65 (0.19)	2.99 (0.25)	0.0634	2.76 (0.21)	2.99 (0.20)	0.3933	275 (0.35)	2.96 (0.32)	0.3594

*Adjusted *p*-values of Tukey’s *post hoc* tests following two-way ANOVA.

Italic values with # are *p*-values of non-parametric t-tests between WT and *Fmr*1 KO mice.

Data are presented as mean (SD).

**TABLE 5:** Latencies of each wave and interpeak in response to click and tone stimuli in WT and *Fmr1* KO mice at P60.

Latency	Click			8 kHz			16 kHz			32 kHz		
	**WT** **(*n* = 10)**	***Fmr1* KO** **(*n* = 12)**	*p* *	**WT** **(*n* = 10)**	***Fmr1* KO** **(*n* = 12)**	***p***	**WT** **(*n* = 10)**	***Fmr1*** **KO** **(*n* = 11–12)**	***p***	**WT** **(*n* = 8–10)**	***Fmr1* KO** **(*n* = 12)**	***p***
Wave I	1.33 (0.05)	1.34 (0.07)	>0.9999 *0.8712^#^*	1.69 (0.10)	1.80 (0.17)	0.0485	1.38 (0.07)	1.52 (0.13)	0.0057 *0.0020^#^*	1.30 (0.05)	1.37 (0.09)	0.3860
Wave II	2.31 (0.06)	2.27 (0.09)	0.9820	2.53 (0.18)	2.57 (0.21)	0.9476	2.10 (0.21)	2.20 (0.19)	0.5097	2.09 (0.17)	2.13 (0.17)	0.9612
Wave III	3.20 (0.13)	3.12 (0.16)	0.8612	3.4 (0.25)	3.46 (0.22)	0.9441	3.03 (0.27)	3.19 (0.22)	0.2611	2.94 (0.15)	2.97 (0.20)	0.9936
Wave IV	4.01 (0.15)	3.93 (0.23)	0.9468	4.12 (0.31)	4.13 (0.27)	>0.9999	3.84 (0.30)	3.89 (0.28)	0.9857	3.59 (0.16)	3.79 (0.35)	0.3613
Interpeak I-II	0.98 (0.06)	0.94 (0.09)	0.9372	0.84 (0.14)	0.78 (0.08)	0.7123	0.72 (0.16)	0.68 (0.12)	0.9295 *0.6840^#^*	0.79 (0.17)	0.76 (0.19)	0.9854
Interpeak II-III	0.89 (0.13)	0.85 (0.13)	0.9687	0.88 (0.19)	0.89 (0.16)	0.9998 *0.3196^#^*	0.93 (0.24)	0.99 (0.15)	0.9020	0.85 (0.23)	0.84 (0.14)	0.9998
Interpeak III-IV	0.81 (0.05)	0.81 (0.10)	>0.9999	0.72 (0.19)	0.67 (0.16)	0.9177	0.81 (0.19)	0.73 (0.13)	0.7034	0.64 (0.16)	0.81 (0.23)	0.0708
Interpeak I-IV	2.67 (0.10)	2.60 (0.17)	0.7775	2.43 (0.29)	2.33 (0.24)	0.8798 *0.3048^#^*	2.46 (0.29)	2.38 (0.25)	0.6911	2.30 (0.15)	2.42 (0.30)	0.9040

*Adjusted *p* values of Tukey’s *post hoc* tests following two-way ANOVA.

Italic values with # are *p*-values of non-parametric t-tests between WT and *Fmr*1 KO mice.

Data are presented as mean (SD).

**TABLE 6:** Latencies of each wave and interpeak in response to click and tone stimuli in cKO and their control mice at P14.

Latency	Click			8 kHz			16 kHz			32 kHz		
	**Control** **(*n* = 14)**	**cKO (*n* = 16)**	*p* *	**Control** **(*n* = 14)**	**cKO** **(*n* = 16)**	***p***	**Control** **(*n* = 14)**	**cKO** **(*n* = 16)**	***p***	**Control (*n* = 13)**	**cKO** **(*n* = 16)**	***p***
Wave I	1.56 (0.14)	1.59 (0.06)	0.9816 *0.1149^#^*	2.28 (0.18)	2.32 (0.11)	0.8898	1.85 (0.18)	1.93 (0.13)	0.3897	1.87 (0.19)	1.94 (0.16)	0.6461 *0.1202^#^*
Wave II	2.75 (0.19)	2.84 (0.16)	0.8708	3.47 (0.32)	3.52 (0.26)	0.9667	3.07 (0.37)	3.20 (0.28)	0.5969	2.90 (0.28)	3.01 (0.26)	0.7832 *0.3656^#^*
Wave III/IV	3.96 (0.33)	4.04 (0.17)	0.9509	4.72 (0.36)	4.88 (0.34)	0.6128	4.40 (0.42)	4.51 (0.39)	0.8441	4.24 (0.32)	4.50 (0.40)	0.2373
Interpeak I-II	1.19 (0.17)	1.24 (0.17)	0.8543 *0.4652^#^*	1.19 (0.20)	1.21 (0.17)	0.9995	1.23 (0.22)	1.27 (0.17)	0.9514	1.03 (0.14)	1.07 (0.19)	0.9755 *0.9203^#^*
Interpeak II-III/IV	1.21 (0.27)	1.21 (0.23)	>0.9999	1.25 (0.15)	1.35 (0.12)	0.4583	1.33 (0.15)	1.31 (0.19)	0.9995	1.34 (0.16)	1.49 (0.19)	0.1980
Interpeak I-III/IV	2.40 (0.22)	2.45 (0.14)	0.9528	2.45 (0.22)	2.56 (0.24)	0.5543	2.55 (0.26)	2.57 (0.27)	0.9958 *0.9104^#^*	2.37 (0.22)	2.56 (0.30)	0.1908

*Adjusted *p*-values of Tukey’s *post hoc* tests following two-way ANOVA.

Italic values with # are *p*-values of non-parametric t-tests between WT and *Fmr*1 KO mice.

Data are presented as mean (SD).

We first examined the effect of DT injection on hearing ability across DTR and *Fmr1* genotypes. The injection was performed at either P5 or P14, which is expected to result in a near-complete hearing loss before the normal onset of hearing (P5) or after substantial hearing experience (P14), respectively. Age-matched saline injections were used as comparisons. Consistent with the previous report (Tong et al., 2015), a single injection of DT (5 ng/g) at either age resulted in substantial hair cell loss within 5–6 days in DTR and KO:DTR mice. We then verified the consequence on ABRs at P28–35 in response to click stimuli. As expected, both DTR and KO:DTR showed characterized ABR patterns following saline injection but had no detectable ABRs to click stimulus up to 90 dB following DT injection (Figures 1B–D). Consistent with the lack of hDTR expression in WT and *Fmr1* KO mice, DT injection did not alter ABR patterns in these two genotypes as compared to saline injection. Thus, we divided the injected mice into four experimental groups based on their *Fmr1* genotype and hearing ability: (1) *WT control group* with FMRP expression and intact afferent inputs, which included DT-injected WT mice and saline-injected DTR mice; (2) *DTR group* with FMRP expression and afferent deprivation, which included DT-injected DTR mice; (3) *Fmr1 KO control group* with FMRP loss and intact afferent inputs, which included DT-injected *Fmr1* KO mice and saline-injected KO:DTR mice; and (4) *KO:DTR group* with FMRP loss and afferent deprivation, which included DT-injected KO:DTR mice.

With DT/saline injection at P5, we then compared the VCN neuron number of the four animal groups at P28, at which progressive cell death in the VCN was complete (Tong et al., 2015). WT and *Fmr1* KO control groups had comparable neuron numbers (*p* = 0.805, parametric Student’s t-test), indicating that the absence of FMRP does not lead to neuronal loss when afferent activity is intact (Figures 1E, F). The DTR group had less neurons than the WT controls, confirming that hair cell deletion at this age induces neuronal loss with FMRP expression (*p* < 0.0001, two-way ANOVA followed by Tukey’s *post-hoc* tests, Figures 1E, F). Two-way ANOVA revealed no significant interaction between *Fmr1* genotype and afferent condition (*p* = 0.588), demonstrating that FMRP absence does not have a significant effect on afferent deprivation-induced neuronal loss in the VCN at pre-hearing age.

**FIGURE 2:**
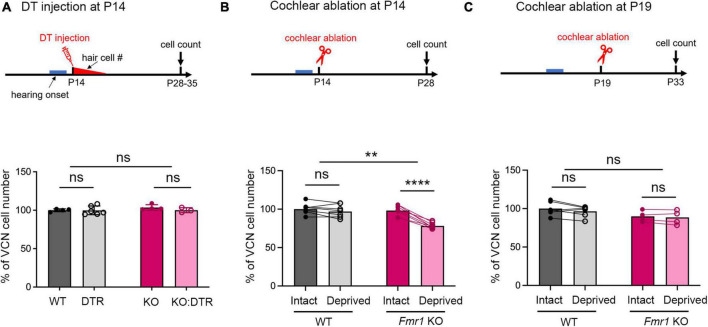
Delayed closure of the critical period for VCN neuronal loss in response to afferent deprivation in *Fmr1* KO mice. (A–C) Experimental timelines (top panel) and quantitative analyses of the neuron number in the VCN (lower panel) across three sets of experiments. (A) Following DT/saline injection at P14, neither DTR nor KO:DTR mice showed significant neuronal loss as compared to WT (WT, *n* = 4; DTR, *n* = 6, *p* > 0.999) and *Fmr1* KO (*Fmr1* KO, *n* = 4; KO:DTR, *n* = 3, *p* = 0.713) controls, respectively (two-way ANOVA followed by Tukey’s *post hoc* tests). (B) Following unilateral cochlear ablation at P14, WT mice did not show significant difference in the neuron number between the deprived (ipsilateral) side and intact (contralateral) side of the VCN (*n* = 7, *p* = 0.315), while *Fmr1* KO mice showed significant neuronal loss on the deprived side compared to the interact side of VCN (*n* = 8, *p* < 0.0001, repeated measures two-way ANOVA followed by Tukey’s *post hoc* tests). (C) Following unilateral cochlear ablation at P19, neither WT nor *Fmr1* KO mice showed a significant difference in the neuron number between the deprived side and intact side of the VCN. ****p < 0.0001; **p < 0.01, ns, not significant.

To examine whether the neuronal susceptibility to deafferentation is diminished in older *Fmr1* KO mice, we next compared the VCN neuron number at P28–P35 across the four experimental groups with DT/saline injection at P14, at which afferent deprivation does not cause VCN cell death in WT (Mostafapour et al., 2000). Neither the DTR group nor the KO:DTR group differed in neuron number when compared to WT and *Fmr1* KO control groups, respectively (WT vs. DTR, *p* > 0.9999; *Fmr1* KO vs KO:DTR, *p* = 0.712, two-way ANOVA followed by Tukey’s *post-hoc* tests, Figure 2A). Thus, afferent deprivation at a post-hearing age does not affect VCN neuron number regardless of *Fmr1* genotype. Together, our results demonstrate that the critical period for neuronal loss in response to afferent deprivation is preserved in *Fmr1* KO mice, and within this critical period, the degree of neuronal loss remains unchanged.

### Critical period for VCN neuronal loss was prolonged in *Fmr1* KO mice

A distinct feature of neuronal susceptibility in the VCN is the abrupt closure of neuronal cell death in response to afferent deprivation. In WT mice, substantial neuronal loss occurs when afferent activity is deprived at and before P11 but not at and after P14, defining the critical period closure between P11–14 (Mostafapour et al., 2000). We thus examined whether FMRP absence affects the time window of the critical period. Because DT-induced hair cell deletion takes about 5–6 days (Tong et al., 2015), we used cochlea ablation as a means of inducing immediate cessation of cochlear inputs to the VCN. We conducted unilateral ablation of the left cochlea in P14 WT and *Fmr1* KO mice and compared the neuron number in the left, afferent-deprived VCN to the right, afferent-intact VCN, which served as a within-animal control. Repeated measures two-way ANOVA revealed a significant interaction between *Fmr1* genotype and afferent condition (*p* < 0.0001). WT mice showed comparable neuron numbers between the left and right VCNs (*p* = 0.315), consistent with P14 being beyond the normal critical period. In contrast, *Fmr1* KO mice had less neurons in the left, afferent-deprived VCN than the right VCN (*p* < 0.0001, repeated measures two-way ANOVA followed by Tukey’s *post-hoc* tests, Figure 2B), indicating that the critical period had not closed at P14 in *Fmr1* KO mice.

We next asked how long the critical period closure is delayed without FMRP. The observation from DT injection at P14, which led to complete hair cell deletion by ∼P19, suggested that this delay is within 5 days. Indeed, following cochlea ablation at P19, *Fmr1* KO mice showed no neuronal loss in the afferent-deprived VCN, undifferentiable from the WT (WT: intact vs. deprived, *p* = 0.174; *Fmr1* KO: intact vs deprived, *p* = 0.708, two-way ANOVA followed by Tukey’s *post-hoc* tests, Figure 2C). Thus, FMRP loss delays the critical period closure in the VCN on a scale of days.

### *Fmr1* KO did not affect afferent deprivation-induced neuronal shrinkage in the VCN

**FIGURE 3:**
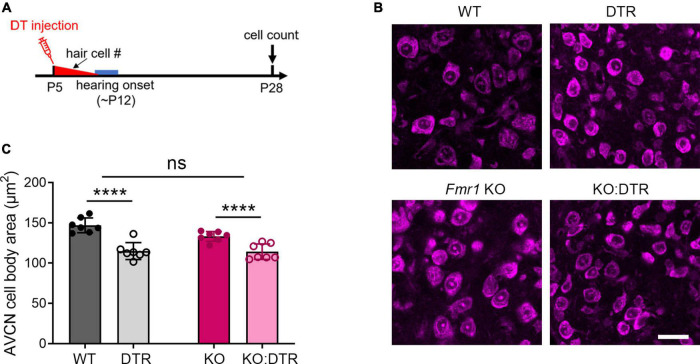
Neuronal cell size was reduced in the AVCN following afferent deprivation in both WT and *Fmr1* KO mice. (A) Experimental timeline for hair cell deletion-induced afferent deprivation. DT or saline was administered at P5 and data analyses were conducted at P28. (B) NeuroTrace-labeling of AVCN neurons. (C) Quantitative analysis of neuronal cell size in the AVCN of four experimental groups at P28 (*n* = 7 per group). Hair cell deletion reduced neuronal cell size in both DTR and KO:DTR groups as compared to WT and *Fmr1* KO controls, respectively (WT vs. DTR, *p* < 0.0001; *Fmr1* KO vs. KO:DTR, *p* < 0.0001, two-way ANOVA followed by Tukey’s *post hoc* tests). There was no significant interaction between *Fmr1* genotype and afferent condition (*p* = 0.0696). Scale bar = 25 μm in panel (B). ****p < 0.0001; ns, not significant.

In addition to cell death, afferent deprivation leads to smaller cell body sizes regardless of the age it is induced (Mostafapour et al., 2000; Tong et al., 2015). We examined whether FMRP loss affects this neuronal size dynamic in the anterior VCN (AVCN) at P28 following DT/saline injection at P5. AVCN neurons were smaller in the *Fmr1* KO control group than the WT control group (*p* = 0.0075, parametric Student’s *t*-test), consistent with previous reports (Rotschafer et al., 2015; Wang et al., 2018). The cell size was reduced in the DTR group as compared to WT controls (*p* < 0.0001, two-way ANOVA followed by Tukey’s *post-hoc* tests, Figure 3), confirming that hair cell deletion induces neuronal shrinkage when FMRP expression is normal. Similarly, the KO:DTR group had reduced cell size as compared to *Fmr1* KO controls (*p* < 0.0001, two-way ANOVA followed by Tukey’s *post hoc* tests). There was no significant interaction between *Fmr1* genotype and afferent condition [*F* (1, 24) = 3.609, *p* = 0.0696, two-way ANOVA]. Since the data in the KO:DTR group did not pass the normality test (*p* = 0.04), we additionally performed non-parametric *t*-test and found no significant change between KO:DTR and DTR groups (*p* = 0.805). This result is consistent with the two-way ANOVA result. To summarize, both FMRP absence and afferent deprivation caused cell shrinkage in the AVCN; however, FMRP absence didn’t affect the degree of afferent deprivation-induced cell shrinkage.

### Hearing onset was delayed and temporally correlated with the critical period closure in *Fmr1* KO mice

**FIGURE 4:**
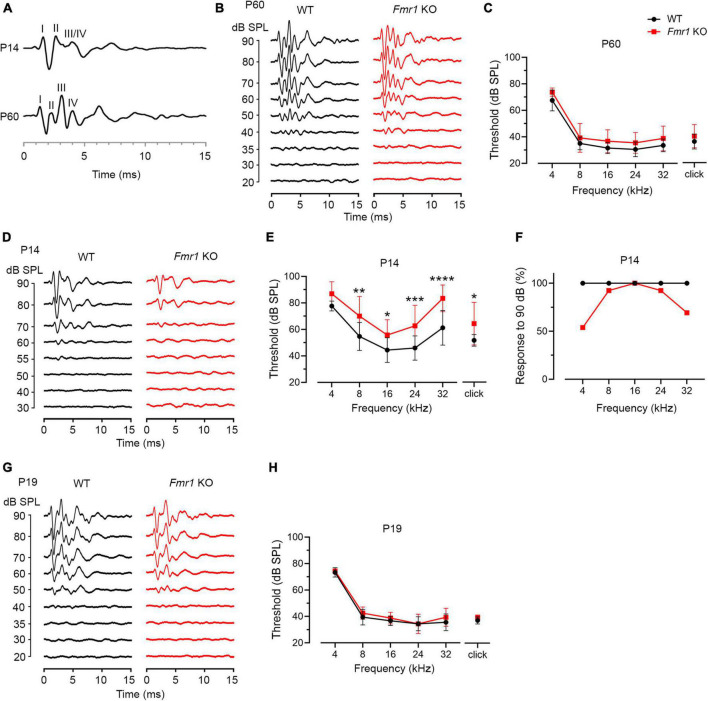
*Fmr1* KO mice displayed a delay in hearing onset. (A) Representative ABR patterns in response to click stimulus at 90 dB in P14 and P60 WT mice. The first three and the first four waves were identifiable at P14 and P60, respectively. (B) Representative ABRs in response to click stimuli at various sound levels at P60. (C) Quantitative analyses of ABR thresholds in WT and *Fmr1* KO mice at P60. There was no significant difference in ABR thresholds between the two genotypes across all stimuli examined (WT, *n* = 10; *Fmr1* KO, *n* = 12, two-way ANOVA followed by Tukey’s *post hoc* tests). (D) Representative ABRs in response to click stimuli at various sound levels at P14. (E) Quantitative analyses of ABR thresholds in WT and *Fmr1* KO mice at P14. *Fmr1* KO mice showed increased ABR thresholds compared to WT mice in response to click and tone bursts of 8, 16, 24, and 32 kHz (WT [*n* = 15–16] vs. *Fmr1* KO [*n* = 13-14]: click, *p* = 0.0124; 8 kHz, *p* = 0.0018; 16 kHz, *p* = 0.0040; 24 kHz, *p* = 0.0005; 32 kHz, *p* < 0.0001, two-way ANOVA followed by Tukey’s *post hoc* tests). The variation in the sample size across frequencies was due to either that some frequencies were tested in a subset but not all animals or that some ABR peaks were not unambiguously identifiable. (F) Percentages of mice responsive to 90 dB SPL across 4-32 kHz frequency stimuli at P14. All WT mice showed ABRs at 90 dB across the frequency range. Only a proportion of *Fmr1* KO mice had ABRs at 90 dB for 4 kHz (7 in 13 animals), 8 kHz (12 in 13 animals), 24 kHz (12 in 13 animals), and 32 kHz (9 in 13 animals) tone bursts. (G) Representative ABRs in response to click stimuli at various sound levels at P19. (H) Quantitative analyses of ABR thresholds in WT and *Fmr1* KO mice at P19. There was no significant difference in ABR thresholds between the two genotypes across all stimuli examined (WT, *n* = 9; *Fmr1* KO, *n* = 8, two-way ANOVA followed by Tukey’s *post hoc* tests). ****p < 0.0001; ***p < 0.001; **p < 0.01; *p < 0.05.

In WT mice, the critical period for VCN neuronal loss closes between P11 and P14. The onset of hearing takes places during the same period (around P12–14), with the full response bandwidth present at P14 (Sprenkle et al., 2001; Song et al., 2006). To examine whether the beginning of substantial auditory experience is associated with the critical period closure in the VCN, we examined how hearing sensitivity is altered in *Fmr1* KO mice at P14 and P60, a mature age. ABR patterns recorded from P60 WT mice were comparable to previous studies (Song et al., 2006; Scimemi et al., 2014), and contained four clearly identifiable waveforms (waves I, II, III, and IV) followed by waves V and VI (Figure 4A). Wave I represents the evoked activity from the SG and auditory nerve, while waves II–IV reflect the responses generated from the brainstem and midbrain (Scimemi et al., 2014; Akil et al., 2016). In response to either click or tone bursts of 4, 8, 16, 24, and 32 kHz, ABR thresholds were comparable between WT and *Fmr1* KO mice at P60 (all *p* > 0.05, two-way ANOVA followed by Tukey’s *post-hoc* tests, Figures 4B, C), consistent with a previous report in the same mouse strain (Chawla and McCullagh, 2021). Non-parametric *t*-tests confirmed no significant changes in ABR thresholds to either click or tones in *Fmr1* KO mice (all *p* > 0.05).

P14 mice showed an immature ABR pattern. Waves I and II were readily identifiable, often followed by a broad wave at the approximate location of adult waves III and IV (Figure 4A). This is consistent with a previous report that a distinct wave IV appears at P15 (Song et al., 2006). We named this broad wave III/IV. At P14, ABR thresholds were increased in *Fmr1* KO mice as compared to WT mice in response to either click or tone stimuli (click, *p* = 0.0124; 8 kHz, *p* = 0.0018; 16 kHz, *p* = 0.0040; 24 kHz, *p* = 0.0005; 32 kHz, *p* < 0.0001, two-way ANOVA followed by Tukey’s *post hoc* tests, Figures 4D, E). Non-parametric *t*-tests confirmed increased ABR thresholds to click and tones in *Fmr1* KO mice (click, *p* = 0.0109; 4 kHz, *p* = 0.0012; 8 kHz, *p* = 0.0062; 16 kHz, *p* = 0.0125; 24 kHz, *p* = 0.0010; 32 kHz, *p* < 0.0001). The response bandwidth was narrowed in *Fmr1* KO mice, with less animals responsive at 90 dB across tone frequencies (Figure 4F). Thus, hearing onset was delayed without FMRP.

If there is a causal relationship between the onset of acoustic-driven activity and critical period closure in the VCN, we would expect a temporal coincidence between the two events under both WT and *Fmr1* KO conditions. We tested this possibility by examining ABR thresholds at P19 when the critical period has closed in *Fmr1* KO mice. In response to either click or tone bursts, ABR thresholds are comparable between the two genotypes (all *p* > 0.05, two-way ANOVA followed by Tukey’s *post-hoc* tests, Figures 4G, H). Non-parametric *t*-tests confirmed no significance changes in ABR thresholds to either click or tones in *Fmr1* KO mice (all *p* > 0.05). Together, the onset of hearing is delayed in *Fmr1* KO mice, coinciding with the critical period closure for VCN neuronal loss.

### ABR wave I displayed a prolonged latency in both developing and mature *Fmr1* KO mice

**FIGURE 5:**
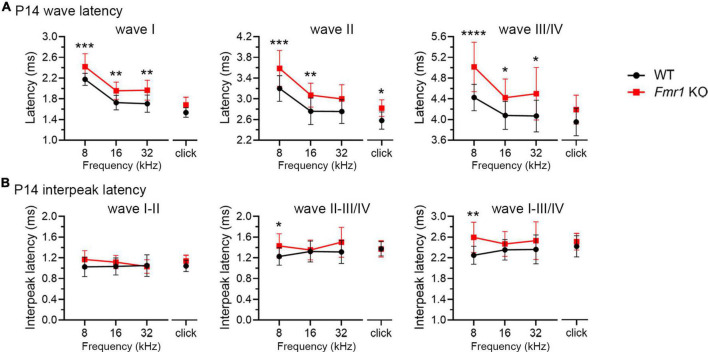
Prolonged latencies of ABR wave I in developing *Fmr1* KO mice (P14). (A) Quantitative analyses of waveform latencies in WT and *Fmr1* KO mice at P14. As compared to WT, *Fmr1* KO mice showed longer latencies of: wave I in response to tone bursts of 8, 16, and 32 kHz frequencies (8 kHz, *p* = 0.0005; 16 kHz, *p* = 0.0014; 32 kHz, *p* = 0.0017); wave II in response to click and tone bursts of 8 and 16 kHz frequencies (click, *p* = 0.0478; 8 kHz, *p* = 0.0003, 16 kHz, *p* = 0.0051); and wave III/IV in response to tone bursts of 8, 16, and 32 kHz frequencies (8 kHz, *p* < 0.0001; 16 kHz, *p* = 0.0373; 32 kHz, *p* = 0.0196, two-way ANOVA followed by Tukey’s *post hoc* tests). (B) Quantitative analyses of interpeak latencies in WT and *Fmr1* KO mice at P14. *Fmr1* KO mice showed longer interpeak latencies of waves II-III/IV (*p* = 0.0394) and waves I-III/IV in response to 8 kHz frequency (*p* = 0.0012, two-way ANOVA followed by Tukey’s *post hoc* tests). Animal numbers for these analyses were seen in Table 3. ****p < 0.0001; ***p < 0.001; **p < 0.01; *p < 0.05.

As described above, individual ABR waves reflect summed neuronal activity along different stages of the ascending auditory pathway. We next attempted to identify potential cellular locations affected by FMRP absence by assessing individual ABR waves. We measured the latencies and interpeak latencies of ABR waves as indicators of signal transmission along the auditory pathway. At P14, the latencies were delayed for all ABR waves (I, II, and III/IV) in *Fmr1* KO mice in response to tone stimuli of 8, 16, and 32 kHz (two-way ANOVA followed by Tukey’s *post-hoc* tests, Figure 5A and Table 3). However, the interpeak latencies (I–II, II–III/IV, and I–III/IV) were largely unchanged, except for a small increase in II–III/IV and I–III/IV interpeak latencies following 8 kHz stimulation (two-way ANOVA followed by Tukey’s *post-hoc* tests, Figure 5B and Table 3). As a few data sets in this analysis fail to pass a normality test, we additionally performed non-parametric tests between *Fmr1* KO and WT with matched stimulation and tone frequency and confirmed similar results (Table 3). Together, these analyses suggest an initial delay at wave I, which progresses to waves II and III/IV.

**FIGURE 6:**
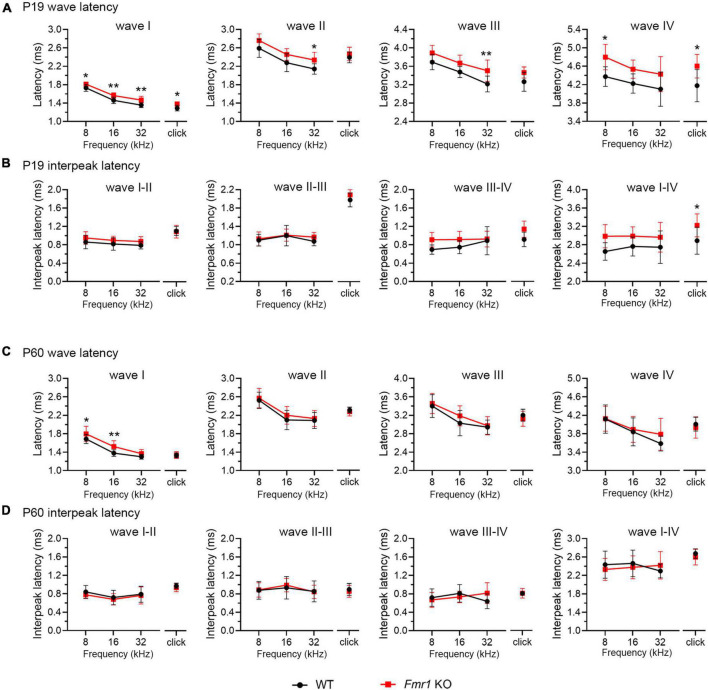
Prolonged latencies of ABR wave I in P19 and P60 *Fmr1* KO mice. **(A)** Quantitative analyses of waveform latencies in WT and *Fmr1* KO mice at P19. *Fmr1* KO mice showed longer latencies of: wave I in response to all stimuli examined (click, *p* = 0.0146; 8 kHz, *p* = 0.0389; 16 kHz, *p* = 0.0048; 32 kHz, *p* = 0.0024); wave II in response to 32 kHz frequency (*p* = 0.044); wave III in response to 32 kHz frequency (*p* = 0.0043); and wave IV in response to click and 8 kHz frequency stimuli (click, *p* = 0.0151; 8 kHz, *p* = 0.0232, two-way ANOVA followed by Tukey’s *post hoc* tests). **(B)** Quantitative analyses of interpeak latencies in WT and *Fmr1* KO mice at P19. *Fmr1* KO mice showed longer interpeak latencies of waves I-IV in response to click stimulus (*p* = 0.0441, two-way ANOVA followed by Tukey’s *post hoc* tests). **(C)** Quantitative analyses of waveform latencies in WT and *Fmr1* KO at P60. *Fmr1* KO mice showed longer latencies of wave I in response to 8 and 16 kHz frequencies (8 kHz, *p* = 0.0485; 16 kHz, *p* = 0.0057, two-way ANOVA followed by Tukey’s *post hoc* tests). **(D)** Quantitative analyses of interpeak latencies in WT and *Fmr1* KO mice at P60. There was no significant difference in interpeak latencies between the two genotypes. Animal numbers for these analyses were seen in Tables 4, 5. **p < 0.01; *p < 0.05.

A delay in wave I latency was also present in P19 *Fmr1* KO mice (two-way ANOVA followed by Tukey’s *post-hoc* tests, Figure 6A and Table 4). The delays in the subsequent waves were diminished, with statistical delay only found in waves II and III in response to 32 kHz tones and in wave IV in response to click and 8 kHz tones. Interpeak latency analyses revealed no genotype-dependent changes, except for a prolonged latency from wave I to wave IV in response to clicks (Figure 6B). At P60, *Fmr1* KO mice continued to have a prolonged wave I latency in response to 8 and 16 kHz (Figure 6C). No alterations in the latency of subsequent waves were identified, nor in any interpeak latencies examined (two-way ANOVA followed by Tukey’s *post hoc* tests, Figures 6C, D and Table 5). Together, these analyses identified an early-onset and long-lasting latency delay in wave I of *Fmr1* KO mice and provided a possible link between peripheral alterations and central deficits (delayed critical period closure).

### Selective *Fmr1* KO from the cochlea led to a prolonged critical period for neuronal loss in the VCN

**FIGURE 7:**
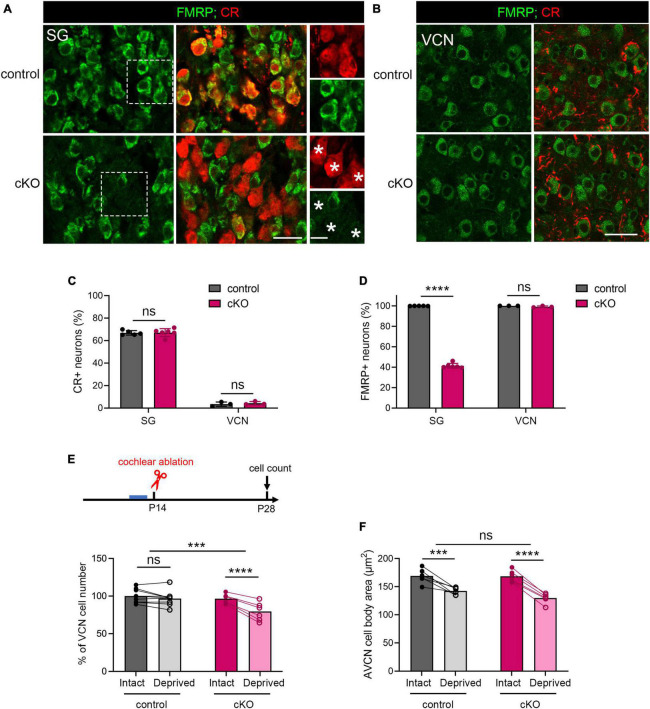
The critical period closure for VCN neuronal loss was delayed in *Fmr1* cKO mice with selective FMRP deletion in the SG but not in the VCN. **(A)** Immunostaining of FMRP, CR, and DAPI in the SG of cKO mice and littermate controls. The right column is the magnification of the boxes in the left column. Stars indicate CR + neurons without FMRP expression in the cKO group. Scale bar = 20 μm (left two columns) and 10 μm (right column). **(B)** Immunostaining of FMRP and CR in the VCN of cKO mice and littermate controls. Scale bar = 20 μm. **(C)** Quantitative analysis of the percentage of CR + neurons in the SG and VCN. In the SG, the percentage of CR + neurons was 60-70% and comparable between cKO and control mice (control, 66.85% ± 2.04%, *n* = 5; cKO, 67.08% ± 3.57%, *n* = 6, *p* = 0.905, Student’s *t*-test). In the VCN, the percentage of CR + neurons was 1-6% and comparable between cKO and control mice (control, 3.44% ± 1.91%, *n* = 3; cKO, 4.26% ± 1.51%, *n* = 3, *p* = 0.592, Student’s *t*-test). **(D)** Quantitative analysis of the percentage of FMRP + neurons in the SG and VCN. In the SG, cKO mice had significantly fewer neurons with FMRP expression compared to control mice (control, 99.9% ± 0.14%, *n* = 5; cKO, 41.29% ± 2.42%, *n* = 6, *p* < 0.0001, Student’s *t*-test). In the VCN, nearly all neurons were FMRP + in both cKO and control mice (control, 100%; cKO, 99.07% ± 0.84%, *n* = 3 per group, (*p* = 0.194, Student’s *t*-test). **(E)** Experimental timeline and quantitative analysis of the neuron number in the VCN of cKO and control mice. Following unilateral cochlea ablation at P14, control mice show no significant difference in the neuron number between the deprived and intact sides of the VCN (*n* = 9, *p* = 0.200). cKO mice showed significant neuronal loss in the deprived side compared to the intact side of the VCN (*n* = 6, *p* < 0.0001, repeated measures two-way ANOVA followed by Tukey’s *post hoc* tests). **(F)** Quantitative analysis of neuronal cell size in the AVCN in cKO and control mice following unilateral cochlear ablation at P14 (*n* = 6 per group). Afferent deprivation resulted in smaller cell size in both cKO and control groups in the deprived side compared to the intact side, respectively (control, *p* = 0.0003; cKO, *p* < 0.0001, repeated measures two-way ANOVA followed by Tukey’s *post hoc* tests). There was no significant interaction between genotype and afferent condition (*p* = 0.0902). ****p < 0.0001; ***p < 0.001; ns, not significant.

A long-standing question pertaining afferent-regulated neuronal dynamics is relative contribution of presynaptic vs. postsynaptic components. We next investigated the site of FMRP action in afferent-regulated VCN neuronal susceptibility. Defective ABR wave I in *Fmr1* KO mice led us to hypothesize that FMRP loss from the cochlea is a key contributor. To examine this hypothesis, we developed a mouse strain with conditional *Fmr1* KO in the SG but not in the auditory brainstem. In mice, gene expression of *Calb2*, which encodes calcium-binding protein CR, is abundant in the SG but not significant in any major auditory brainstem cell groups (Chiba et al., 2006; Lee et al., 2017). Crossing a *Fmr1^loxp^* line with a CR-iCre line driven by the endogenous *Calb2* promoter/enhancer elements generated selective *Fmr1* KO in the SG but not in the VCN. In the SG, the percentage of CR+ neurons was comparable between control (66.87%) and cKO (67.08%) mice (*p* = 0.905, parametric Student’s *t*-test, Figures 7A, C). This is consistent with a previous single-cell RNA sequencing report that *Calb2* is identified in 69% mouse Type I SGNs (Petitpre et al., 2018). While almost all SGNs were FMRP+ in controls (99.9%), only 41.29% were FMRP+ in cKO mice (Figures 7A, D), meaning that about 60% SGNs in cKO mice lost FMRP expression (*p* < 0.0001, parametric Student’s t-test). As expected, FMRP deletion was restricted to CR+ neurons (asterisks in Figure 7A). In the brain, CR+ cell bodies were rare in the VCN (control, 3.42%; cKO, 3.53%, *p* = 0.592, parametric Student’s *t*-test) (Figures 7B, C). Cytoplasmic FMRP immunoreactivity was identified in nearly all VCN neurons in cKO mice (99.07%), undifferentiable from controls (100%, *p* = 0.194, parametric Student’s t-test, Figures 7B, D). In summary, cKO mice had selective FMRP deletion in the presynaptic, but not the postsynaptic, neurons of the SG-VCN circuit.

We then performed unilateral cochlea ablation in cKO mice and their littermate controls at P14 and assessed the neuron number in the VCN at P28-30 (Figure 7E). Similar to WT, control mice showed comparable neuron numbers between the two VCNs, indicating a closed critical period (*p* = 0.200, repeated measures two-way ANOVA followed by Tukey’s *post-hoc* tests). cKO mice had fewer neurons in the afferent-deprived VCN as compared to the afferent-intact VCN of the same animals (*p* < 0.0001), demonstrating an open critical period at this age. Repeated measures two-way ANOVA revealed a significant genotype × afferent condition interaction (*p* = 0.0006). These results demonstrate that selective FMRP loss from SGNs delays the critical period closure for VCN neuronal loss.

On the other hand, both cKO and control mice showed significant and comparable reduction in the cross-sectional cell body area in the afferent-deprived AVCN (control, *p* = 0.0003; cKO, *p* < 0.0001, repeated measures two-way ANOVA followed by Tukey’s *post hoc* tests, Figure 7F), indicating no effect of cochlear FMRP loss in this neuronal dynamic. Interestingly, in the afferent-intact AVCN, the neuronal size was comparable between cKO and control mice (*p* = 0.6332, parametric Student’s *t*-test), in contrast to a significant cell size reduction in global *Fmr1* KO mice (Figure 3; Rotschafer et al., 2015). This result demonstrates that the maintenance of neuronal size in the AVCN requires FMRP cell-autonomous function.

### Hearing onset is largely normal in cKO mice with selective FMRP loss in the SG

**FIGURE 8:**
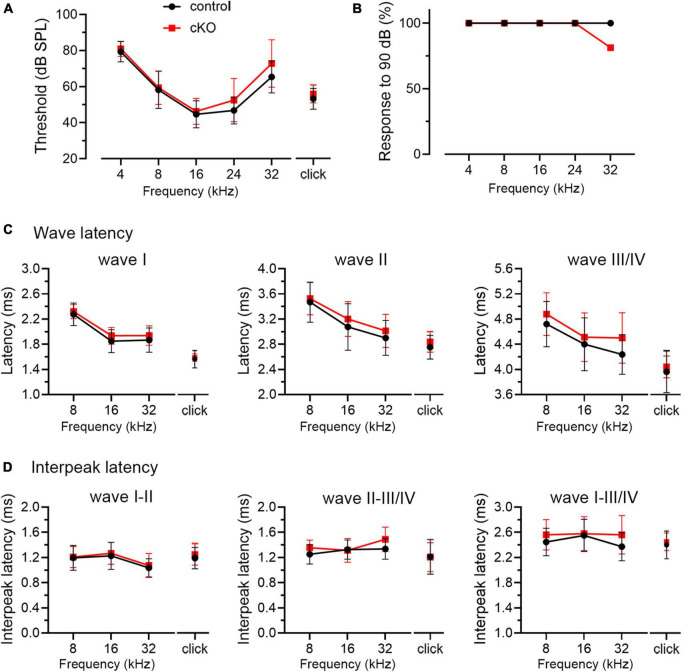
ABR threshold and waveform latency were largely intact in *Fmr1* cKO mice with selective FMRP deletion in the SG but not in the VCN. **(A)** Quantitative analyses of ABR thresholds in cKO and control mice at P14. There was no significant difference in ABR thresholds between the two genotypes across all stimuli examined (control, *n* = 14–16; cKO, *n* = 16–18, two-way ANOVA followed by Tukey’s *post hoc* tests). **(B)** Percentages of mice responsive to 90 dB across 4–32 kHz frequency stimuli at P14. All control mice (100%) and only a subpopulation of cKO mice (13 in 16 animals, 81.25%) showed ABR signal at 90 dB in 32 kHz. **(C,D)** Quantitative analyses of waveform latencies and interpeak latencies in cKO and control mice at P14. There was no significant difference in waveform latencies and interpeak latencies between the two genotypes examined (two-way ANOVA followed by Tukey’s *post hoc* tests). Animal numbers for panels **(C,D)** were seen in Table 6.

Delayed hearing onset and altered wave I latency in *Fmr1* KO mice (Figures 4, 5) suggest a potential role of peripheral FMRP in regulating auditory processing. We compared ABR thresholds and latencies between cKO and their littermate controls at P14. We found no significant difference in the threshold, latency, and interpeak latency of each ABR wave in response to any stimulus examined between the two genotypes (two-way ANOVA followed by Tukey’s *post-hoc* tests, Figures 8A, C, D and Table 6). Non-parametric *t*-tests confirmed no significance changes in ABR thresholds to either click or tone stimuli in cKO mice (all *p* > 0.05). However, while all control mice responded to all frequency tones at 90 dB, only 81.25% of cKO mice (13 in 16 animals) were responsive to 32 kHz frequency at 90 dB (Figure 8B).

## Discussion

In this study, we identified specific alterations in the temporal signature of a critical period in a mouse model of FXS. In the VCN, the closure of the critical period for deafferentation-induced neuronal loss was delayed. This phenomenon was highly selective among other dynamic properties of VCN neurons and was temporally correlated with the presence of substantial acoustic-driven signals. Using a conditional genetic approach, we determined a peripheral contribution to this central deficit, revealing a novel mechanism for the generation of sensory dysfunction in FXS.

One important finding of our study is the identification of a delay in the closure of the critical period for deafferentation-induced neuronal loss in the VCN of *Fmr1* KO mice. Intriguingly, the degree of neuronal loss was not affected when deafferentation occurred within the critical period. This indicates that FMRP is required for timely closing the window period for neurons being responsive, but probably not an irreplaceable signal underlying neuronal response. This selectivity was further supported when AVCN cell shrinkage, one type of deafferentation-induced neuronal response that does not have a critical period (Sie and Rubel, 1992; Mostafapour et al., 2000; Tong et al., 2015), was independent of FMRP. A preference for FMRP regulation on the temporal aspect of critical periods was also observed in the somatosensory cortex. A delay in the time window for synaptic plasticity (long-term potentiation), but not the degree of the potentiation, was found in *Fmr1* KO mice (Harlow et al., 2010). Interestingly, lesion-induced somatosensory map plasticity was not altered in either the degree of change within the critical period or the timing of critical period closure (Harlow et al., 2010). This is in contrast with other reports that tone exposure-induced plasticity in the tonotopic organization was diminished in the auditory cortex (Kim et al., 2013) and visual deprivation-induced downregulation of synaptic inhibition was absent in the visual cortex of *Fmr1* KO mice (Zhong et al., 2018). These variations between brain regions and types of critical period suggest that FMRP regulates neuronal dynamic events across sensory modalities in context-specific manners.

Multiple mechanisms may contribute to this specialized FMRP function. One is the tightly regulated FMRP level in the developing brain. In postnatal mouse brains, for example, FMRP level is high within the first 1–2 weeks, followed by a rapid (within several days) and dramatic decline in the third week (Lu et al., 2004; Wang et al., 2004; Harlow et al., 2010; Cook et al., 2011; Pacey et al., 2013). This age-dependent reduction of FMRP control may facilitate the closure of critical periods. Additionally, FMRP expression and subcellular localization are dynamically regulated by changes in afferent activity in several cell types including auditory neurons (Irwin et al., 2000, 2005; Todd and Mack, 2000; Gabel et al., 2004; Yu et al., 2021), providing another potential regulatory mechanism. In addition to the dynamics of FMRP itself, the observed delay in the critical period closure may be due to a general developmental delay when FMRP is absent. Examples include delays in dendritic and synaptic pruning, glutamatergic signal maturation, and GABA polarity switch from depolarizing to hyperpolarizing (Harlow et al., 2010; Till et al., 2012; He et al., 2014). Last, but equally important, FMRP may directly regulate the molecular signals that are involved in defining the time window of critical periods. In the case of VCN, for example, microglial activation and immune response have been implicated in regulating the critical period for deafferentation-induced neuronal death (Zhao and Lurie, 2004; Harris et al., 2008), and FMRP is known to inhibit pro-inflammatory and phagocytic response in microglia (Hodges et al., 2020; Parrott et al., 2021).

On the other hand, the maintenance of neuronal cell body size in the AVCN displays a multi-faced relationship with FMRP. First, afferent deprivation-induced cell shrinkage does not appear to be FMRP-dependent. We observed comparable degrees of cell size reduction across wildtype, global Fmr1 KO, and Fmr1 cKO mice. Second, with intact afferent inputs (no deprivation), neuron cross-sectional area is reduced in the AVCN of global Fmr1 KO but not cKO mice in which FMRP expression is normal in AVCN neurons, suggesting a cell-autonomous requirement of FMRP in maintaining cell size. It is unknown whether this requirement is cell-type specific. The VCN contains heterogeneous cell populations including bushy cell (globular and spherical bushy) and stellate cell (D-stellate and T-stellate) (Oertel, 1991; Rubio, 2018). They are similarly innervated by the auditory nerve and other synaptic inputs, but with differential physiological properties and ascending projection targets (Friauf and Ostwald, 1988; Cant and Benson, 2003; Campagnola and Manis, 2014; Heeringa et al., 2018; Rubio, 2018). In this study, although we did not differentiate cell types, a major portion of the cells included in the cell size analysis is presumably globular and spherical bushy cells based on the location of these cells (see Tierney et al., 1997). Future studies with physiologically identified VCN cell types are needed to address this question.

A second contribution of this study is the identification of a peripheral contribution to FXS, showing a similar delay in the critical period closure following selective FMRP loss in SGNs as that observed in *Fmr1* KO mice. The inner ear has not been studied as a pathological site in FXS, probably due to largely normal hearing sensitivity in FXS individuals (McCullagh et al., 2020). However, the recent discovery of FMRP expression in the cochlea (Wang et al., 2022) and the well-documented peripheral influence on the development and function of the central auditory system (Rubel and Fritzsch, 2002; Polley et al., 2013; Ryugo, 2015; Lesicko and Llano, 2017) raised the possibility of an involvement of peripheral FMRP in cochlear and brain development. In the mouse cochlea, FMRP is intensively expressed in SGNs both during development and after maturity (Wang et al., 2022). Our results in ABR analyses indicate that prolonged latencies likely arise in the SG and auditory nerve in *Fmr1* KO mice. In the avian brainstem, autonomous FMRP reduction in auditory neurons resulted in altered synaptic development and weakened neurotransmission (Wang et al., 2018), supporting the idea that FMRP loss from SGNs may similarly damage signal transmission to the brainstem. In non-sensory systems, presynaptic FMRP is also known as a regulator of synaptic formation and neurotransmission, which subsequently affect intrinsic excitability of the postsynaptic target neurons (Wang et al., 2023). Notably, given that CR has been identified as a neuronal marker of type Ia SGNs (Petitpre et al., 2018; Shrestha et al., 2018; Sun et al., 2018; Wang et al., 2021), our results may further suggest a primary contribution of this SGN subtype in FMRP-mediated closure of a VCN critical period. Together, our study provides the first direct evidence supporting a peripheral contribution to FXS auditory pathogenesis.

This finding is supported by two important studies of genetic models of autism spectrum disorders (ASD) although not directly in FXS. Selective deletion of the X-linked methyl-CpG-binding protein 2 (*Mecp2*) gene, a key genetic cause of Rett Syndrome, in peripheral somatosensory neurons results in aberrant tactile sensitivity by disrupting GABAergic presynaptic inhibition to the CNS (Orefice et al., 2016). Treating the *Mecp2* KO mice with a peripherally restricted GABA_A_ receptor agonist reduces tactile sensitivity and improves ASD-related phenotypes (Orefice et al., 2019). In the case of FXS, peripherally somatosensory neurons normally express FMRP and *Fmr1* KO mice display abnormal tactile sensitivity (Orefice et al., 2016). Although not examined, it is likely that the periphery mechanism of FXS spans across sensory modalities including at least auditory and somatosensory systems.

Finally, our results provide a better understanding of the relationship between the onset of sensory-driven activity and brain critical periods. In *Fmr1* KO mice, the failure of the critical period for VCN neuronal loss to close at P14 concomitant with increased ABR thresholds lend credence to the idea that auditory critical period closure is associated with the onset of substantial auditory experience. In further support of this idea, both hearing sensitivity and the critical period closure resume WT-like conditions within the same time period (P14-19). However, the observations from the cKO mice suggest that the relationship, if one exists, may be complex. In this mouse line, hearing onset is largely normal, but the critical period closure is delayed. Several possibilities may account for this unexpected finding. First, the link between acoustic-driven afferent activity and critical period closure may occur at the cellular level. Signal transmission may be altered in the 60% of SGNs lacking FMRP in cKO mice and in turn responsible for the delay in the critical period closure. The remaining 40% of SGNs with FMRP may be sufficient to maintain normal ABRs, which reflects synchronized activity. Second, instead of being casually related, hearing onset and the critical period closure may be triggered by a common event that is present in WT and *Fmr1* KO mice but lost in cKO mice. For example, intact FMRP control in the auditory brainstem may create an imbalanced efferent modulation to the FMRP-deficient cochlea in cKO mice, while this effect may be counteracted in *Fmr1* KO mice.

In conclusion, the involvement of cochlear FMRP expression in shaping the temporal signatures of the critical period for afferent-regulated neuronal susceptibility in auditory brainstem neurons reveals a novel peripheral mechanism underlying auditory dysfunction in FXS. The importance of peripheral FMRP in brain development is likely common across sensory modalities, given that FMRP expression is also identified in the vertebrate retina (Frederikse et al., 2015; Guimaraes-Souza et al., 2016). Future investigations of peripheral FMRP functions in sensory organs and their central regulation are expected to facilitate our understanding of sensory pathogenesis in neurodevelopmental diseases and shed light on therapy development.

## Data availability statement

The raw data supporting the conclusions of this article will be made available by the authors, without undue reservation.

## Ethics statement

This animal study was reviewed and approved by Florida State University Institutional Animal Care and Use Committee.

## Author contributions

XY: experimental design, data acquisition and analysis, and writing manuscript. YW: experimental design and writing manuscript. Both authors contributed to the article and approved the submitted version.
